# Darcy–Forchheimer Magnetized Nanofluid flow along with Heating and Dissipation Effects over a Shrinking Exponential Sheet with Stability Analysis

**DOI:** 10.3390/mi14010106

**Published:** 2022-12-30

**Authors:** Liaquat Ali Lund, Abdul Fattah Chandio, Narcisa Vrinceanu, Ubaidullah Yashkun, Zahir Shah, Ahmed Alshehri

**Affiliations:** 1 KCAET Khairpur Mirs, Sindh Agriculture University, Tandojam 70060, Sindh, Pakistan; 2 Department of Electronic Engineering, Quaid-E-Awam University of Engineering, Science & Technology Nawabshah, Nawabshah 67480, Sindh, Pakistan; 3 Faculty of Engineering, Department of Industrial Machines and Equipments, “Lucian Blaga” University of Sibiu, 10 Victoriei Boulevard, 550024 Sibiu, Romania; 4 Department of Mathematics and Social Sciences, Sukkur IBA University, Sukkur 79165, Sindh, Pakistan; 5 Department of Mathematical Sciences, University of Lakki Marwat, Lakki Marwat 28420, Khyber Pakhtunkhwa, Pakistan; 6 Department of Mathematics, Faculty of Sciences, King Abdulaziz University, Jeddah 21589, Saudi Arabia

**Keywords:** Darcy–Forchheimer, nanofluid, viscous dissipation, joule heating, duality, stability

## Abstract

Nanoparticles have presented various hurdles to the scientific community during the past decade. The nanoparticles dispersed in diverse base fluids can alter the properties of fluid flow and heat transmission. In the current examination, a mathematical model for the 2D magnetohydrodynamic (MHD) Darcy–Forchheimer nanofluid flow across an exponentially contracting sheet is presented. In this mathematical model, the effects of viscous dissipation, joule heating, first-order velocity, and thermal slip conditions are also examined. Using similarity transformations, a system of partial differential equations (PDEs) is converted into a set of ordinary differential equations (ODEs). The problem is quantitatively solved using the three-step Lobatto-three formula. This research studied the effects of the dimensionlessness, magnetic field, ratio of rates, porosity, Eckert number, Prandtl number, and coefficient of inertia characteristics on fluid flow. Multiple solutions were observed. In the first solution, the increased magnetic field, porosity parameter, slip effect, and volume percentage of the copper parameters reduce the velocity field along the η-direction. In the second solution, the magnetic field, porosity parameter, slip effect, and volume percentage of the copper parameters increase the η-direction velocity field. For engineering purposes, the graphs show the impacts of factors on the Nusselt number and skin friction. Finally, the stability analysis was performed to determine which solution was the more stable of the two.

## 1. Introduction

Flow across a Darcy-medium has extensive uses and considerable value in medicinal, chemical, and contemporary ecological frameworks. Numerous processes, including oil applications in different processes, thermal management in geothermal exchange formats, atomic head waste processes, water development, and water purification, are included. The classic model of Darcy is comprise the non-Darcian porous medium, which is a constrained form of this medium for the aforementioned applications and operations. The term Darcy’s Law clarifies the fluid movement through a porous medium. This rule is applicable in scenarios with low porosity and low velocity. Forchheimer showed the velocity square factor in Darcy’s velocity equation to analyze the boundary and inertia features. Muskat and Meres [1] referred to this as the Forchheimer word using Maxwell nanofluid flow through an isothermally heated stretching sheet. Rasool et al. [2] explained the effect of of compressibility and uniform porosity affect heat transfer in Maxwell nanofluid flow through an isothermally heated stretching sheet. Seddeek [3] studied the flow of Darcy–Forchheimer under the impact of thermophoresis and dissipation over a vertical surface. Ahmed et al. [4] demonstrate the behavior of magnetized gyrotactic microorganisms in the flow of Eyring–Powell nanofluid with Darcy–Forchheimer and a thermal radiation effect. They found that “motile density profiles are deprecated by higher values of the bioconvective Lewis number and Peclet number”. Hydromagnetic flow with variable nonuniform source/sink and viscosity on a porous surface has been studied by Pal and Mondal [5], who advocated for the theory of Darcy–Forchheimer. Turkyilmazoglu [6] investigated the properties of time-dependent magnetized flow using a revolving permeable disk with varying viscosity. Khan and Alzahrani [7] offer modeling and numerical simulation for convective radiative flow using the Darcy–Forchheimer and second-order velocity slip equations. According to Nagaraju et al. [8], an impulsive porous unsteady flow of liquid flow was seen to occupy the space over a stretched surface.

Choi [9] initially established the concept of nanofluids after conducting observational studies on various nanoparticle suspensions in carrier fluids. Nanofluids may be produced by suspending nanoparticles such as metallic oxides, metals, nitrides, metal carbides, and carbon nanotubes in working fluids such as ethylene, oils, glycol, and water. The incorporation of nanoparticles into carrier liquids improves their thermophysical characteristics. Nanofluids have essential uses in a variety of scientific and technological domains, including mechanical cooling, illness treatment, diagnostic testing, chemical processes, heat exchangers, atomic reactors, microfluidics, and others [10]. Alotaibi and Eid [11] examined the MHD Brownian diffusion and thermophoresis effects on nanofluid flow and heat transmission on a stretching surface and found that “the velocity profile dwindled with augmented values of the magnetic and Forchheimer parameters”. Qayyum et al. [12] investigate how joule heating, activation energy, and dissipation affect the slip flow of a Prandtl–Eyring nanofluid. Bang and Chang [13] looked at how heat transfer increased when water-based nanofluids passed over a simple surface in fresh water. Khan et al. [14] looked at a nanofluid made of silver and water and found that the silver nanofluid was a better conductor and moved heat well. Several noteworthy works on nanofluids are provided in the following references [15,16,17,18,19]. Daniel et al. [20] investigated a solution for nanofluid flow stretching when the MHD electrical effect is executed with ohmic heating.

The theory of magnetohydrodynamics (MHD) outlines how magnetic fields affect the way that nanofluids flow. MHD has several practical uses, including in nuclear reactor cooling, MHD generators, cancer treatment, plasma research, oil exploration, crystal fiber manufacture, paper production, geothermal energy extraction, and boundary layer flow management. The impact of chemical reactions on heat transfer performance in Williamson nanofluid MHD flows within a porous medium is investigated by Alrihieli et al. [21]. Kumaran et al. [22] described the chemically reactive MHD flow of Maxwell and Casson nanoliquids with a heat source/sink. Turkyilmazoglu [23,24] revealed the unsteady 2D flow behavior of MHD nano liquid flow by impulsively rotating permeable disks. This behavior was seen in the flow of the nanoliquid. The MHD flow of a micropolar nanofluid on a stretched surface with joule, dissipation, and convection heating taking place at the convective boundary was examined by Waqas et al. [25]. The boundary slip mechanisms in the MHD flow of chemically reactive nanomaterials with dissipation were explored by [26]. Nayak et al. [27] scrutinized the characteristics of a three-dimensional MHD nanofluid flow across an exponentially porous stretched surface with convective boundary conditions (BCs). The characteristics of the MHD flow of a non-Newtonian fluid were explored by Sarada et al. [28], who conducted their research across a stretching sheet. Anuar et al. [29] used a stability analysis to investigate the flow of MHD carbon nanotubes across a nonlinearly deforming sheet. The melting effects on the MHD incompressible unsteady Casson flow of a nanoliquid on a stretching plate were considered by Mabood et al. [30]. MHD flow issues in a variety of flow fields have been an interesting subject of discussion by scholars and scientists [31,32,33,34].

The influence of slip conditions on fluid flow have not received a lot of attention, particularly when it comes to nanofluids, as shown by a comprehensive review of the research that has been published. A great number of important fluid applications exhibit slip boundary circumstances, such as the improvement of valves of the heart and cavity interiors, as well as the cleaning of prosthetic valves of the heart [35]. It is important to note that the condition of there being no slippage does not always hold in actual practice. It is possible to provide a straightforward explanation for the slip velocity condition by pointing out that flowing liquids do not have nil velocity with respect to their interaction with the barrier of a solid. Andersson [36] was likely the first person to present the idea of slip impact having an impact on the flow of a boundary layer. Wang et al. [37] looked at what happened to a Maxwell nanofluid when it slipped over a stretch that grew longer and longer at an exponential rate. The researchers discovered that as the slip parameter velocity component was increased, a diminishing behavior was seen in both directions. According to the findings of Saleem and Abd El-Aziz [38], the effect of fluid friction irreversibility decreases near to a surface as the slip parameter increases; nevertheless, farther away in the flow system, the irreversibility of heat transfer has been shown to be a more prominent factor. The unsteady flow on an exponential sheet was examined by Haider et al. [39], who discovered that the slip condition had a diminishing influence on the skin friction coefficient. Imran et al. [40] examined the behavior of an MHD generalized Maxwell fluid when it was applied to an exponentially accelerating infinite vertical surface along with a slip condition. According to Reddy et al. [41], a rise in the slip condition causes the velocity profiles to increase as well.

Joule heating has also been one of the most intriguing impacts to be implemented since joule heating has a significant impact on the MHD flow of fluids. Ohmic or joule heating is the process of converting electrical energy into thermal energy via material resistive losses. In addition, the heating of joule impact is used extensively and experimentally in the majority of electrical and electronic equipment. Reddy et al. [42] described the effect of ohmic heating on the flow of nanofluids along elastic barriers. They explained that the existence of ohmic heating might result in a temperature rise. Maskeen et al. [43] investigated the MHD flow in a Cu-Al_2_O_3_/water hybrid nanofluid via a vertically extending cylinder. Sajid et al. [44] reported a computational solution for the MHD flow of a ferrofluid in a curved channel with semi-porosity and ohmic heating. Patel and Singh [45] considered joule heating in their study of Walters-B liquid flow. Furthermore, Kamran et al. [46], Gholinia et al. [47], and Khan et al. [48] investigated the joule heating effect numerically in the analysis of micropolar fluid, Williamson fluid, and Casson fluid. Khan et al. [49] and Hussain et al. [50] independently examined the application of joule heating to a stretching cylinder for MHD Carreau and MHD Sisko nanofluid flows. Yan et al. [51] examined the MHD Cu-Al_2_O_3_/water flow of a hybrid nanofluid via an exponentially reducing permeable sheet and discovered binary solutions. Khan et al. [52] recently investigated the Eyring–Powell flow of fluids along with ohmic heating and the variable viscosity impacts for the material of the wire coating application.

The current work theoretically studies the MHD Darcy–Forchheimer flow of a water-based nanofluid across an exponentially diminishing surface in the presence of ohmic heating, viscous dissipation, and first-order slip conditions, as inspired by the aforementioned literature. Nanofluids have been thoroughly discussed in the aforementioned literature; thus, a *Cu*–water-based nanofluid was selected as an example to explore the influences of the different physically applied factors. Four aspects of the heat transfer concert of nanofluids are called into question. (i) If no suction is applied, are double solutions of similarity conceivable for flow caused by an exponentially contracting sheet? (ii) Does an increase in the Forchheimer parameter and Eckert (ohmic heating) number cause an interruption in the separation of the boundary layer and a decrease in the rate of heat transfer? (iii) Does the use of a *Cu*–water nanofluid increase the rate of heat transfer compared to a conventional fluid? (iv) Does the increasing volume fraction of copper nanoparticles in a fluid based on water increase the rate of heat transfer? Consequently, this paper will provide answers to each of the preceding questions. Using exponential transformations, the governing model is transformed into ODEs. All computations are performed with a *bvp4c* solver, and, in a few instances, it is attempted to compare the numerical outcomes with the previously published literature. The authors are assured that the current piece of research is novel and will have a substantial impact on future researchers in the field of fluid dynamics.

## 2. Mathematical Description of the Problem

**Figure 1:**
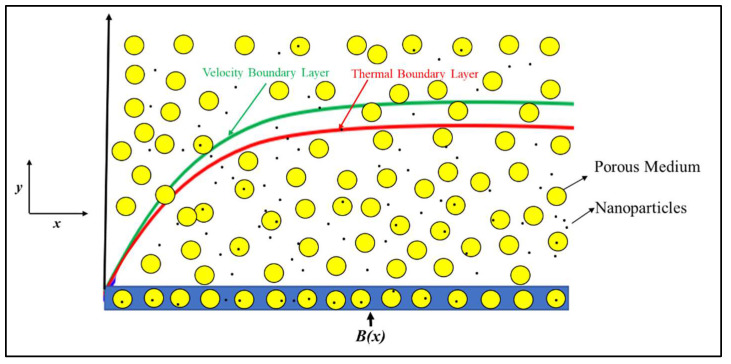
Model of nanofluid flow.

Let us take into account the effects of viscous dissipations, joule heating, and slip conditions in the context of the steady two-dimensional incompressible flow of an electrically conducting nanofluid across a diminishing surface in a porous medium along with Darcy–Forchheimer. The physical model of the considered problem can be seen in Figure 1.

The assumption of a system of cartesian coordinates, in which the *x*-axis is parallel to the contracting surface and the *y*-axis is perpendicular to it, is also taken into account. To the perpendicular of the shrinking sheet, a uniform magnetic field B(x)=B0ex2l is applied with strength, where *B*_0_ is a constant magnetic resilience. The properties of the nanofluid and solid along with water are given in Table 1 and Table 2.

We disregard the induced magnetic field, and, subsequently, the Reynolds number is so very low. The governing equations for a steady nanofluid flow under these conditions are as follows [40,51]:

(1) $$\frac{\partial u}{\partial x}+\frac{\partial v}{\partial y}=0,\;\;\;\;\;$$

(2) $$u\frac{\partial u}{\partial x}+v\frac{\partial u}{\partial y}=\frac{\mu_{nf}}{\rho_{nf}}\frac{\partial^{2}u}{\partial y^{2}}-\frac{\mu_{nf}}{\rho_{nf}}\frac{1}{K}u-\frac{b}{\sqrt{K}}u^{2}-\frac{\sigma B^{2}\left(x\right)u}{\rho_{nf}},$$

(3) $$u\frac{\partial T}{\partial x}+v\frac{\partial T}{\partial y}=\frac{k_{nf}}{{\left(\rho C_{p}\right)}_{nf}}\frac{\partial^{2}T}{\partial y^{2}}+\frac{\mu_{nf}}{{\left(\rho C_{p}\right)}_{nf}}{\left(\frac{\partial u}{\partial y}\right)}^{2}+\frac{\sigma B^{2}u^{2}}{{\left(\rho C_{p}\right)}_{nf}}.$$

The associated BCs related to (1–3) are

(4) $$\{\begin{array}{c} v=v_{w}\;,\;u=u_{w}+B\vartheta_{f}\frac{\partial u}{\partial y},T=\;T_{w}\left(x\right)\;+D\frac{\partial T}{\partial y},\;at\;y=0\;\\ u\;\to \;0,\;\;\;T\;\to T_{\infty },\;as\;\;\;y\;\to \;\infty \end{array}.$$

In these equations *v* and *u* represent components of velocity in the *y*- and *x*-directions, correspondingly, *ρ* denotes the density of the fluid, *σ* is the electrical conductivity of the fluid, *b* is the local inertia coefficient, *K* is the porous medium permeability, *T* is the fluid temperature, and subscript *nf* shows the properties of the nanofluid explained in Table 1. $T_{w}=T_{\infty }+T_{0}e^{\frac{2x}{\ell }}$ is the wall temperature, and *T*_∞_ is the ambient temperature. In addition, vw=−ϑa2lex2l S, where *S* is the injunction/suction parameter, uw=−a exl is the surface velocity, B=B1e−x2l is the velocity slip factor, and D=D1 e−x2l is the thermal slip factor.

These transformations of similarity are used to obtain the corresponding similarity solutions:

(5) ψ=2ϑflaex2lf(η) , θ(η)=(T−T∞)(Tw−T∞), η=ya2ϑfl ex2l.

The stream function *ψ* is expressed in components of velocity as

(6) $$u\;=\;\frac{\partial \psi }{\partial y}\;\;,\;v\;=\;-\;\frac{\partial \psi }{\partial x}.$$

K=2K0 e−xl is used to represent the porous media permeability. By applying Equations (5)–(6) to Equations (1)–(3), the equation of continuity is fulfilled, and the equations of momentum and energy can be expressed as

(7) (f‴−K1f′){(1−∅)+∅(ρsρf)}(1−∅)2+ff″−(2+FS)f′2−Mf′{(1−∅)+∅(ρsρf)}=0,

(8) $$\frac{\frac{k_{nf}}{k_{f}}}{Pr\left\{\left(1-\emptyset \right)+\emptyset \left(\frac{{\left(\rho C_{p}\right)}_{s}}{{\left(\rho C_{p}\right)}_{f}}\right)\right\}}\theta^{\prime \prime }+f\theta^{\prime }-4f^{\prime }\theta +\frac{Ec}{\left\{\left(1-\emptyset \right)+\emptyset \left(\frac{{\left(\rho C_{p}\right)}_{s}}{{\left(\rho C_{p}\right)}_{f}}\right)\right\}{\left(1-\emptyset \right)}^{2}}{\left(f^{\prime \prime }\right)}^{2}\;+\frac{EcM}{\left\{\left(1-\emptyset \right)+\emptyset \left(\frac{{\left(\rho C_{p}\right)}_{s}}{{\left(\rho C_{p}\right)}_{f}}\right)\right\}}{\left(f^{\prime }\right)}^{2}=0,$$

along with the BCs:

(9) $$\{\begin{array}{c} S-f\left(0\right)=0\;,\;\theta \left(0\right)=1+\delta_{T}\theta^{\prime }\left(0\right),\;f^{\prime }\left(0\right)+1=\delta f^{\prime \prime }\left(0\right)\\ \theta \left(\eta \right)\to 0,\;f^{\prime }\left(\eta \right)\;\to \;0\;as\;\eta \to \infty \end{array},$$

where $M=\frac{2l\sigma {\left(B_{0}\right)}^{2}}{\rho a}$ is the Hartmann number, $K_{1}=\frac{l\vartheta_{f}}{aK_{0}}$ is the permeability parameter, $F_{S}=\frac{2lb}{\sqrt{K}}$ is the Forchheimmer parameter, $Pr=\frac{\vartheta_{f}}{\alpha_{f}}$ denotes the Prandtl number, and $Ec=\frac{a^{2}}{T_{0}{\left(C_{p}\right)}_{f}}$ is the Eckert number. Furthermore, $\delta =B_{1}\sqrt{\frac{\vartheta_{f}a}{2l}}$ is the velocity slip, and $\delta_{T}=D_{1}\sqrt{\frac{a}{2\vartheta_{f}l}}$ is the thermal slip parameter. 

The coefficient of skin friction and the local Nusselt number are all relevant physical quantities, and they can be written as

(10) $$C_{f}=\frac{{\left[\mu_{nf}\frac{\partial u}{\partial y}\right]}_{y=0}}{\rho a^{2}},\;N_{u}=\frac{-xk_{nf}{\left(\frac{\partial T}{\partial y}\right)}_{y=0}}{k_{f}\left(T_{w}-T_{\infty }\right)}.$$

By applying Equations (5)–(6) in Equation (10), we obtain

(11) Cf(Rex)12=1(1−∅)2f″(0),Nu(Rex)−12=−(knfkf)θ′(0).

## 3. Linear Stability Analysis

Recently, numerous scholars have examined multiple fluid solutions for various fluid kinds and flow circumstances. From an experimental standpoint, it is worthwhile to determine which solution is physically dependable and applicable. Therefore, linear stability is necessary for validating the dependability of solutions. Merkin [53] and Weidman et al. [54] suggested reducing the controlling boundary layer of Equations (3)–(5) to the following unsteady forms for the stability analysis:

(12) $$\frac{\partial u}{\partial t}+u\frac{\partial u}{\partial x}+v\frac{\partial u}{\partial y}=\frac{\mu_{nf}}{\rho_{nf}}\frac{\partial^{2}u}{\partial y^{2}}-\frac{\mu_{nf}}{\rho_{nf}}\frac{1}{K}u-\frac{b}{\sqrt{K}}u^{2}-\frac{\sigma B^{2}u}{\rho_{nf}},$$

(13) $$\frac{\partial T}{\partial t}+u\frac{\partial T}{\partial x}+v\frac{\partial T}{\partial y}=\frac{k_{nf}}{{\left(\rho C_{p}\right)}_{nf}}\frac{\partial^{2}T}{\partial y^{2}}+\frac{\mu_{nf}}{{\left(\rho C_{p}\right)}_{nf}}{\left(\frac{\partial u}{\partial y}\right)}^{2}+\frac{\sigma B^{2}u^{2}}{{\left(\rho C_{p}\right)}_{nf}},$$

where *t* represents time. Additionally, a novel similarity transformation is presented as

(14) ψ=2ϑflaex2lf(η,τ), θ(η,τ)=(T−T∞)(Tw−T∞),η=ya2ϑfl ex2l , τ=a2lexl·t.

Using Equation (14), Equations (12)–(13) can be written as follows:

(15) (∂3f(η, τ)∂η3−K1∂f(η, τ)∂η){(1−∅)+∅(ρsρf)}(1−∅)2+f(η, τ)∂2f(η, τ)∂η2−(2+FS)(∂f(η, τ)∂η)2−M∂f(η, τ)∂η{(1−∅)+∅(ρsρf)}−∂2f(η, τ)∂τ∂η=0,

(16) $$\frac{\left(\frac{k_{nf}}{k_{f}}\right)\frac{\partial^{2}\theta \left(\eta ,\tau \right)}{\partial \eta^{2}}}{\mathrm{Pr}\left\{\left(1-\emptyset \right)+\emptyset \left(\frac{{\left(\rho C_{p}\right)}_{s}}{{\left(\rho C_{p}\right)}_{f}}\right)\right\}}+\frac{\partial \theta \left(\eta ,\tau \right)}{\partial \eta }f\left(\eta ,\;\tau \right)-4\theta \left(\eta ,\tau \right)\frac{\partial f\left(\eta ,\;\tau \right)}{\partial \eta }\;+\frac{Ec{\left(\frac{\partial^{2}f\left(\eta ,\;\tau \right)}{\partial \eta^{2}}\right)}^{2}}{\left\{\left(1-\emptyset \right)+\emptyset \left(\frac{{\left(\rho C_{p}\right)}_{s}}{{\left(\rho C_{p}\right)}_{f}}\right)\right\}{\left(1-\emptyset \right)}^{2}}+\frac{EcM{\left(\frac{\partial f\left(\eta ,\;\tau \right)}{\partial \eta }\right)}^{2}}{\left\{\left(1-\emptyset \right)+\emptyset \left(\frac{{\left(\rho C_{p}\right)}_{s}}{{\left(\rho C_{p}\right)}_{f}}\right)\right\}}\;-\frac{\partial \theta \left(\eta ,\tau \right)}{\partial \tau }=0,$$

along with the new BCs:

(17) $$\{\begin{array}{c} \;\frac{\partial f\left(0,\;\tau \right)}{\partial \eta }=-1+\delta \frac{\partial^{2}f\left(0,\;\tau \right)}{\partial \eta^{2}},f\left(0,\tau \right)=S,\;\theta \left(0,\tau \right)-\delta_{T}\frac{\partial \theta \left(0,\;\tau \right)}{\partial \eta }=1\\ \frac{\partial f\left(\eta ,\;\tau \right)}{\partial \eta }\;\to \;0,\;\theta \left(\eta ,\tau \right)\;\to \;0,\;\;\;\;\;\;\;\;\;\;as\;\eta \to \infty \end{array}.\;\;\;$$

To test the stability of the steady flow solutions solving the boundary value problem (7–9), where $\theta \left(\eta \right)=\theta_{0}\left(\eta \right)$ and $f\left(\eta \right)=f_{0}\left(\eta \right)$, one can express

(18) $$\{\begin{array}{c} f\left(\eta ,\tau \right)-e^{-\varepsilon \tau }F\left(\eta ,\tau \right)=f_{0}\left(\eta \right)\\ \theta \left(\eta ,\tau \right)-e^{-\varepsilon \tau }G\left(\eta ,\tau \right)=\theta_{0}\left(\eta \right) \end{array},$$

where *f*_0_(*η*) and *θ*_0_(*η*) are minor relatives of *F*(*η*) and *G*(*η*), respectively. Moreover, *ε* represents the unknown eigenvalues. When the eigenvalue problem (16–18) is solved, an endless collection of eigenvalues is obtained. We must choose the least eigenvalue from this set. If the smallest eigenvalue (*ε*) is negative, this shows that the flow is unstable and demonstrates the growth of disruptions and physical impossibility. If the smallest eigenvalue is positive, on the other hand, this indicates that the solution is physically reliable and stable. The following equations result from applying Equation (18) in (15–16):

(19) (F0‴−K1F0′){(1−∅)+∅(ρsρf)}(1−∅)2+f0F0″+F0f0″−2(2+FS)f0′F0′−MF0′{(1−∅)+∅(ρsρf)}+εF0′=0,

(20) $$\frac{\left(\frac{k_{nf}}{k_{f}}\right)G_{0}^{\prime \prime }}{Pr\left\{\left(1-\emptyset \right)+\emptyset \left(\frac{{\left(\rho C_{p}\right)}_{s}}{{\left(\rho C_{p}\right)}_{f}}\right)\right\}}+f_{0}G_{0}^{\prime }+F_{0}\theta_{0}^{\prime }-4f_{0}^{\prime }G_{0}-4F_{0}^{\prime }\theta_{0}\;+\frac{2Ecf_{0}^{\prime \prime }F_{0}^{\prime \prime }}{\left\{\left(1-\emptyset \right)+\emptyset \left(\frac{{\left(\rho C_{p}\right)}_{s}}{{\left(\rho C_{p}\right)}_{f}}\right)\right\}{\left(1-\emptyset \right)}^{2}}+\frac{2EcMf_{0}^{'}F_{0}^{\prime }}{\left\{\left(1-\emptyset \right)+\emptyset \left(\frac{{\left(\rho C_{p}\right)}_{s}}{{\left(\rho C_{p}\right)}_{f}}\right)\right\}}\;+\varepsilon G_{0}=0,$$

Subject to the BCs,

(21) $$\{\begin{array}{c} F_{0}\left(0\right)=0,\;\;\;\;\;\;F_{0}^{\prime }\left(0\right)=\delta F_{0}^{\prime \prime }\left(0\right),\;G_{0}\left(0\right)=\delta_{T}G_{0}^{\prime }\left(0\right)\\ F_{0}^{\prime }\left(\eta \right)\to 0,\;\;\;G_{0}\left(\eta \right)\to 0,\;\;as\;\;\eta \to \infty \end{array}.$$

Conferring with Haris et al. [55], in order to find the stability of (19–21), one boundary condition on $F_{0}^{\prime }\left(\eta \right)$ and $G_{0}\left(\eta \right)$ must be relaxed. In this case, it is important to note that we relaxed $F_{0}^{\prime }\left(\eta \right)\to 0\;as\;\eta \to \infty$ into $F_{0}^{\prime \prime }\left(0\right)=1$. In addition, we fixed all parameters, including $F_{S}=0.5,\;K_{1}=0.5,\;Pr=6.2,Ec=0.2,\;\delta =\delta_{T}=0.1,$ and varied values of *S* and ∅.

## 4. Result and Discussion

The system of PDEs (2–3) is transformed into ODEs (7–8) using exponential transformations (5). The converted ODEs (7–8) are then resolved by employing the *bvp4c* function along with the BCs (9) in MATLAB. A water-based nanofluid along with Darcy–Forchheimer phenomena over an exponentially shrunk sheet under the influence of different physical effects, parameters, the Hartmann number, the Prandtl number, the Eckert number, thermal slip, suction, Forchheimer, velocity slip, and porosity were studied. Before beginning to analyze the outcomes of the problem at hand, it is necessary to validate that the numerical coding is functioning effectively; in this regard, Table 3 is built for comparison with the previously published data by Waini et al. [56] when *Pr =* 6.2, *ϕ* = 0 (regular fluid), $K_{1}=F_{S}=M=Ec=\delta =\delta_{T}=0$, for a stretching surface (i.e., $f^{\prime }\left(0\right)=1$), and a great agreement is observed. Therefore, the existing numerical method and its MATLAB coding are used with full sureness to solve the problem at hand. In addition, we considered fluids at a temperature of 25 °C; therefore, the researchers recommended that the Prandtl number for water at 25 °C be equal to 6.2.

**Figure 2:**
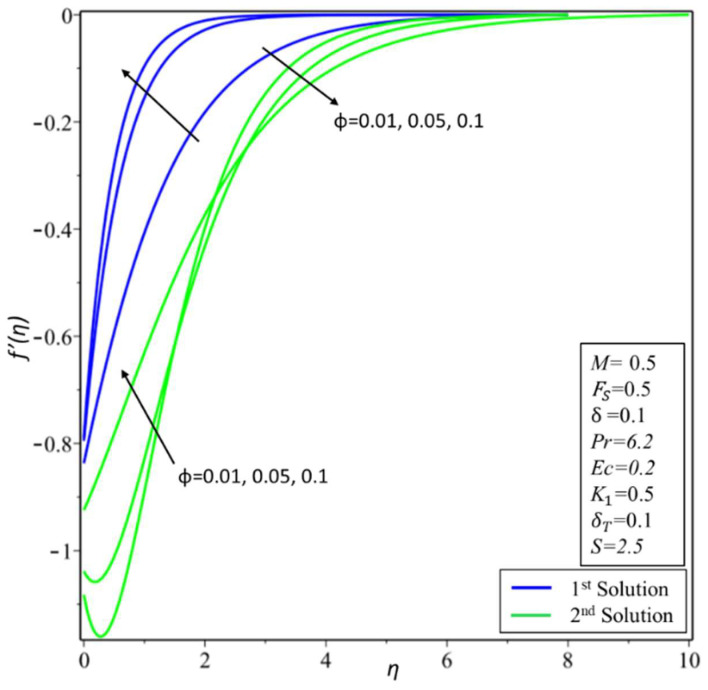
Changes of *f*′(*η*) for ∅.

**Figure 3:**
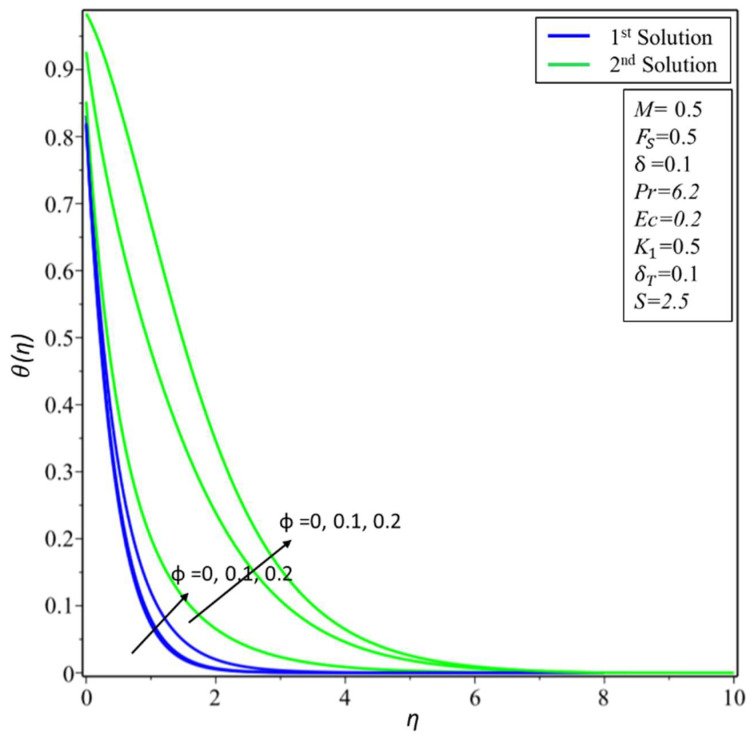
Changes of *θ*(*η*) for ∅.

**Figure 4:**
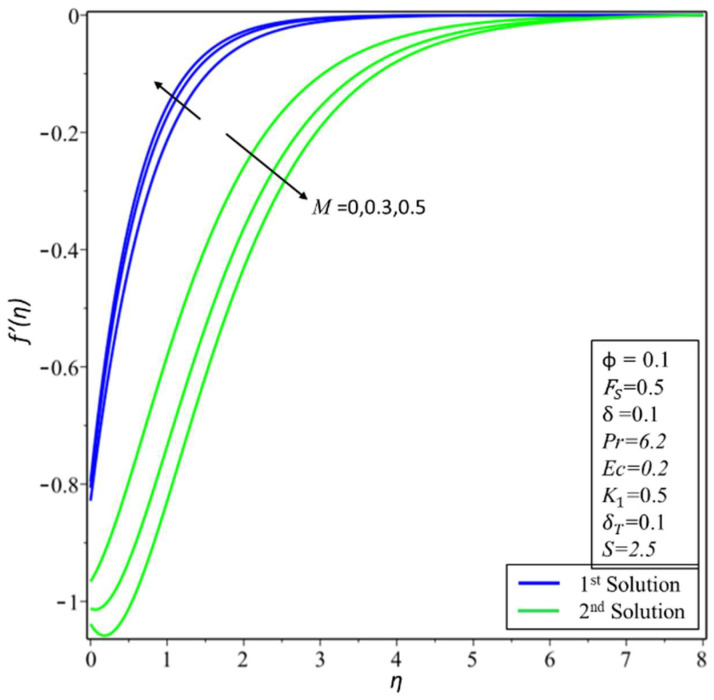
Changes of *f*′(*η*) for *M*.

**Figure 5:**
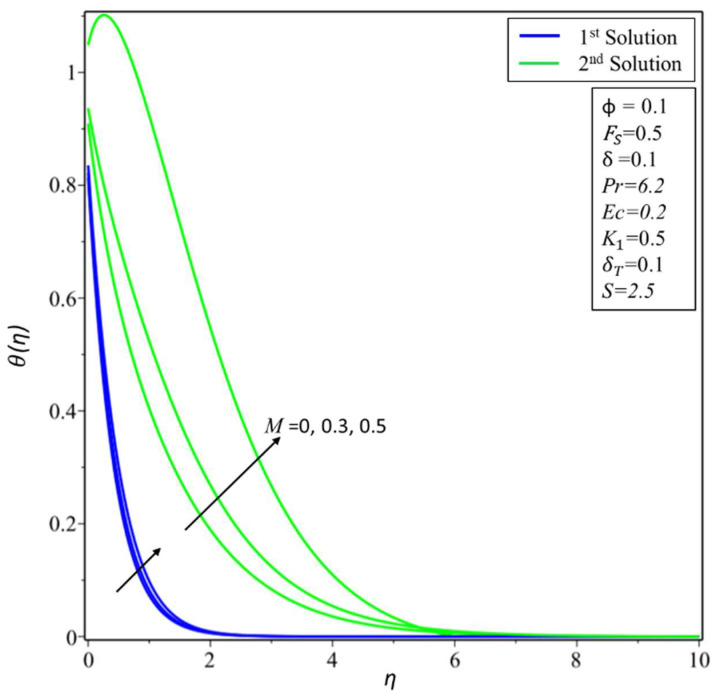
Changes of *θ*(*η*) for *M*.

Figure 2 depicts the variation of the velocity profile *f*′(*η*) for changed values of the volume fraction parameter: *ϕ* = 0.01, 0.05, and 0.1 along different physical parameters. It is observed that the first solution is decreasing; this is because a nanofluid with a high-volume fraction has a lower velocity because a higher number of particles per unit volume means the fluid is more viscous or heavier, requiring more energy to move. On the other hand, the second solution is increasing near the surface for the high-volume fraction. Figure 3 illustrates the results of temperature variations *θ*(*η*) for different values of *ϕ* = 0.01, 0.05, and 0.1. It is observed from the figure that the temperature variation increases for both solutions. In practice, the nanoparticles disperse energy as heat. More nanoparticles produce more energy, raising the temperature and thickness of the thermal boundary layer. Figure 4 presents the variation of *f*′(*η*) for changed estimates of the Hartmann number = 0, 0.3, and 0.5. It is observed that the first solution decreases; it is believed that the application of a transverse magnetic field results in a Lorentz force similar to a drag force, which tends to resist the fluid flow and thus reduces its velocity in the profiles of the first solution. Meanwhile, the second solution has an increasing nature with an increase in the values of *M*. In Figure 5, the temperature variation for different values of *M* = 0, 0.3, and 0.5 is shown. It is noted that the estimates of *θ*(*η*) increase for both solutions with enhancing estimates of *M*. Due to the fact that a Lorentz force can enhance mass transport, which easily induces convective motions and energy transport, the thermal boundary layer thickness increases in both solutions.

**Figure 6:**
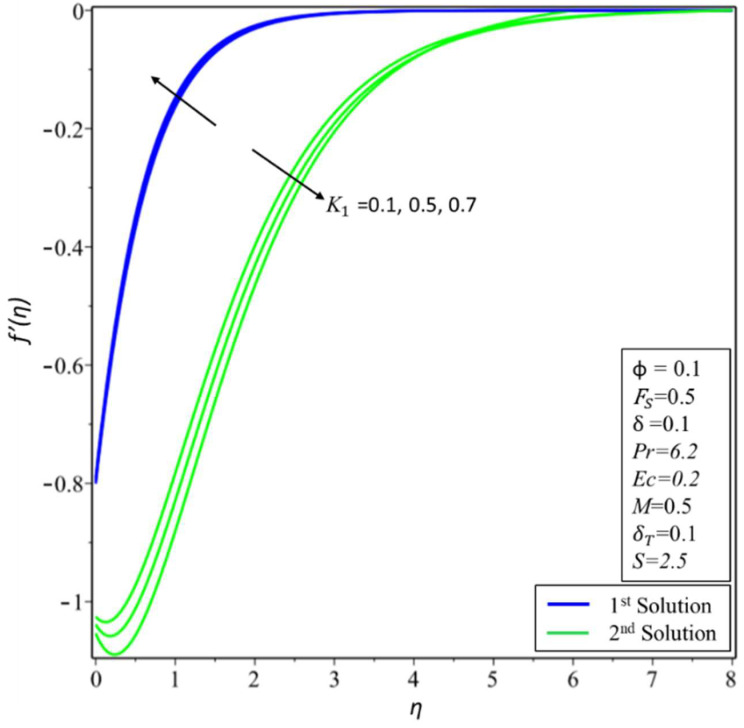
Changes of *f*′(*η*) for *K*_1_.

**Figure 7:**
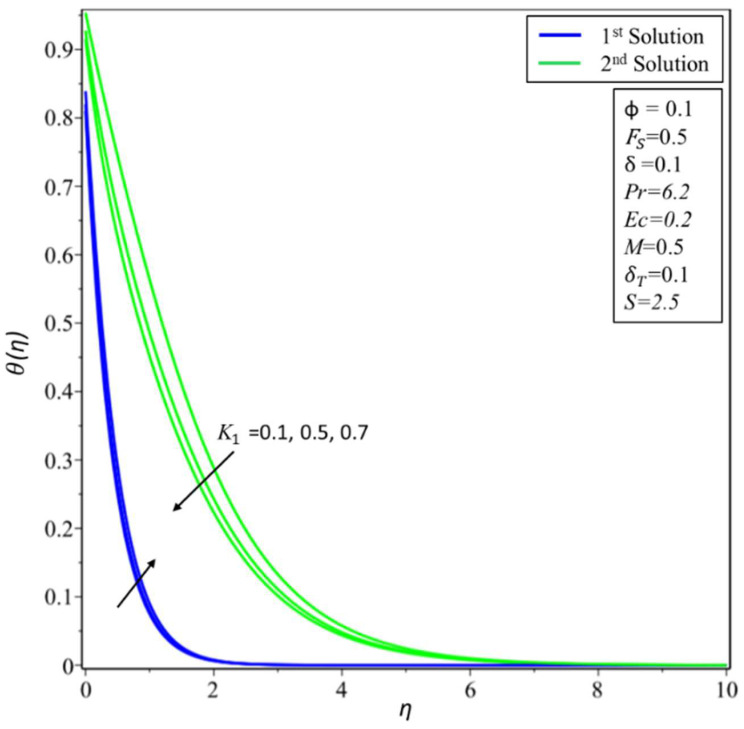
Changes of *θ*(*η*) for *K*_1_.

**Figure 8:**
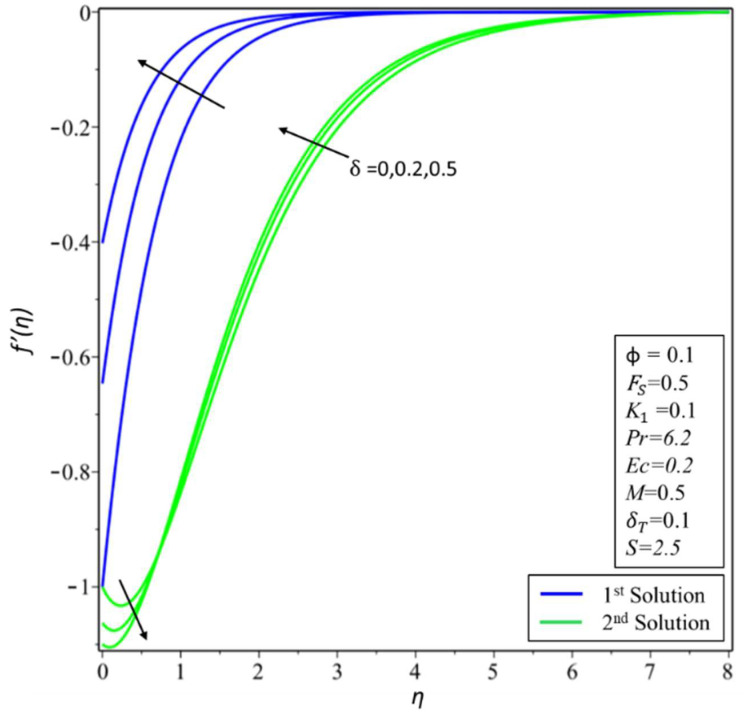
Changes of *f*′(*η*) for *δ*.

**Figure 9:**
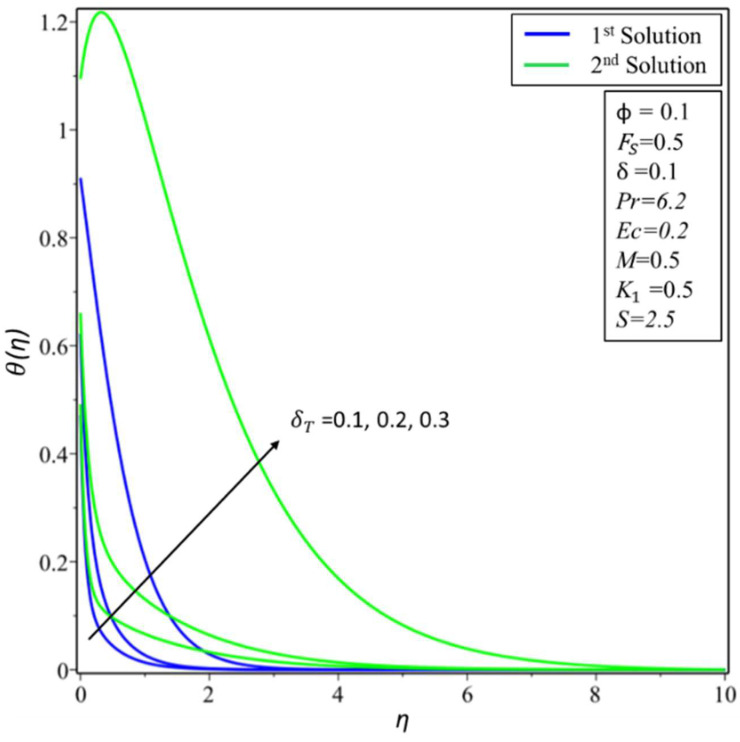
Changes of *θ*(*η*) for *δ_T_*.

Figure 6 and Figure 7 depict the variation of *f*′(*η*) and *θ*(*η*) for various values of permeability *K*_1_. In Figure 6, estimates of *f*′(*η*) are reduced in the first solution; this is because effective density and permeability are directly proportional to each other, and, as shown in Equation (2), high permeability causes the velocity and momentum thickness of the boundary layer to decrease. On the other hand, profiles of velocity are increased in the second solution as the values of *K*_1_ = 0.1, 0.5, and 0.7 rise. In Figure 7, the variation of *θ*(*η*) increases in the first solution and reduces in the second solution. The variation of *f*′(*η*) is presented in Figure 8 for different estimates of the slip parameter: *δ =* 0, 0.2, and 0.5. Here, it shows that the behavior of the first solution is decreasing, but the second solution is increasing initially and then decreasing as the value of *δ* rises. This decreasing behavior of the velocity profiles is due to the velocity slip, which is the difference between the particle velocity and the undisturbed nanofluid velocity at the particle location. The variation of *θ*(*η*) is illustrated in Figure 9 and shows an increase in temperature for both the first and second solutions as *δ_T_* = 0.1, 0.2, and 0.3.

**Figure 10:**
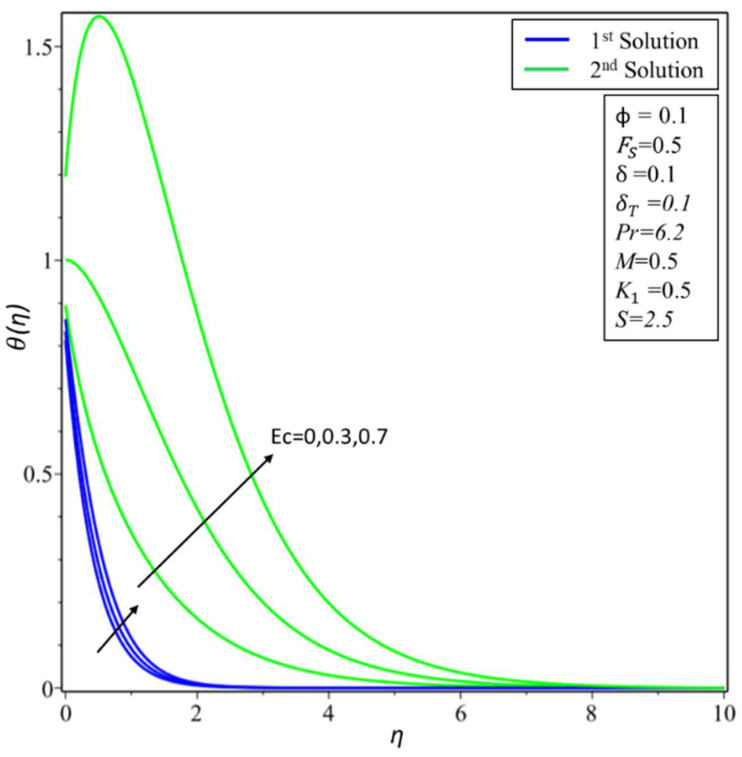
Changes of *θ*(*η*) for *Ec*.

**Figure 11:**
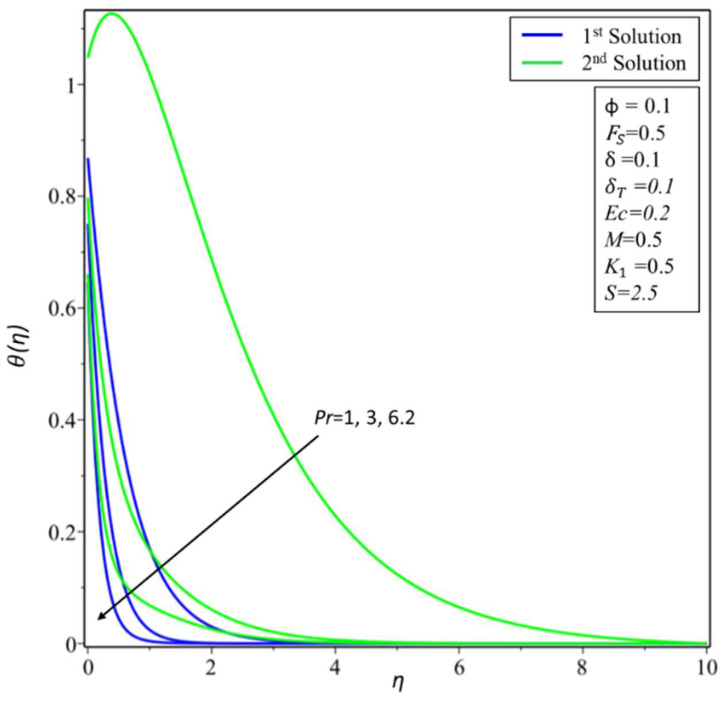
Changes of *θ*(*η*) for *Pr*.

The variation of *θ*(*η*) is shown in Figure 10 for various estimates of the Eckert number: *Ec* = 0, 0.3, and 0.7. An increase in temperature is noticeable for both the second and first solutions, as the *Ec* values increase. Physically, *Ec* describes the enthalpy and kinetic energy connection. Consequently, it minimizes viscous fluid stress by converting kinetic energy into internal energy. A rise in internal energy causes fluid enhancement. The effect of the Prandtl number *Pr* is discussed in Figure 11. It is noticed that as the values of *Pr* = 1, 3, and 6.2 increase, the variations of *θ*(*η*) decrease for both the first and second solutions. The Prandtl parameter has an opposite relationship with the diffusivity of thermal energy. Greater values of *Pr* cause a lower diffusivity of thermal energy. The lower diffusivity of thermal energy brings a decrement in the temperature profiles and is connected with the thermal layer thickness.

**Figure 12:**
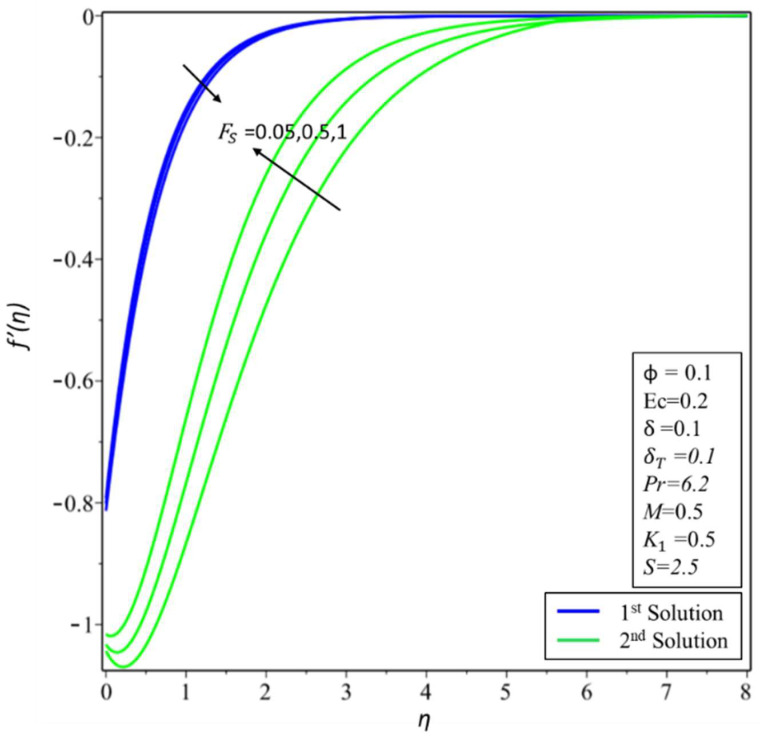
Changes of *f*′(*η*) for *F_s_*.

**Figure 13:**
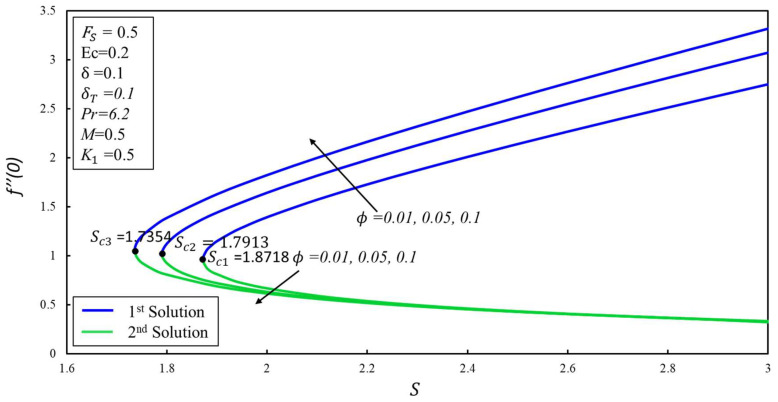
Skin-friction coefficient *f*″(0) against *S* and ∅.

**Figure 14:**
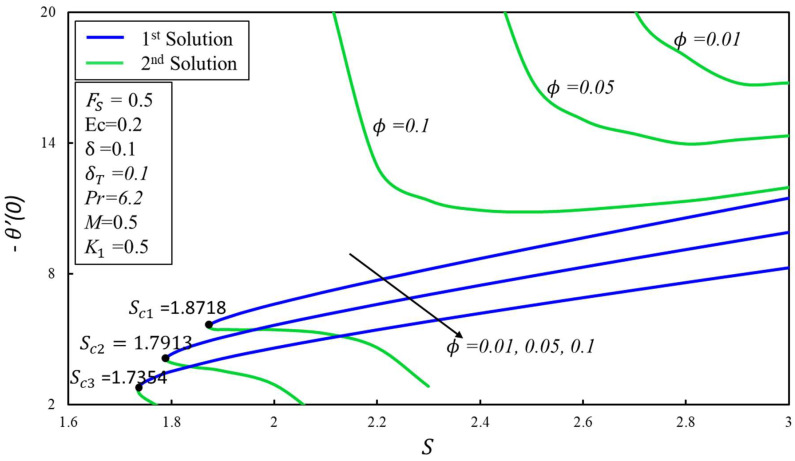
Heat transfer rate −*θ*′(0) against *S* and ∅.

Figure 12 depicts the variation of *f*′(*η*) for various values of Forchheimer *F_s_*. It is noted that the increase in *F_s_* = 0, 0.5, and 1 causes a decrease/increase in the variation of *f*′(*η*) for both the first and second solutions. Physically, an initial drag force is produced due to a rise in *F_s_*, which acts as a barrier for *f*′(*η*) which causes the velocity profile to dissipate. Figure 13 depicts the effect of the skin friction *f*″(0) coefficient against *S* for different values of *ϕ* = 0.01, 0.05, and 0.1. The critical values are *S_ci_* = 1.8718, 1.7913, and 1.7354, respectively, where *i* = 1, 2, and 3. It is noticed that *f*″(0) values rise in the first solution but decrease in the second solution as the values of *ϕ* increase. As the estimates of *ϕ* increase, the critical values decrease. The effect of the Nusselt number, that is, the heat transfer rate −*θ*′(0) against the magnitudes of *S* for various values of *ϕ* = 0.01, 0.05, and 0.1 is depicted in Figure 14. As the value of *ϕ* increases, the value of both solutions decreases. The critical values of *S_ci_* = 1.8718, 1.7913, and 1.7354, where *i* = 1, 2, and 3. The values of *S_ci_* become smaller with increasing values of *ϕ*. Physically, it causes the extension of boundary layer separation. The multiple solutions of −*θ*′(0) are observed for numerous magnitudes of *S_ci_*. The values of −*θ*′(0) decrease with the values of *ϕ* for both solutions. The dual solutions are obtained only as the value of *S* is larger than the critical value of *S_ci_*. There are no similarity solutions outside these critical values (*S* < *S_ci_*).

The fluctuations of the smallest eigenvalues *ε*_1_ against *S* and ∅ are displayed in Table 4 for $F_{S}=0.5,\;K_{1}=0.5,\;Pr=6.2,\;\delta =\delta_{T}=0.1,$ and *Ec* = 0.2. As explained by Equation (18), the flow is steady when the initial disturbance declines with time. This will be the case for *ε*_1_ > 0. In the meantime, the flow for *ε*_1_ < 0 is unstable due to the beginning rise of disturbance as time progresses, $e^{-\varepsilon \tau }\to \infty$ as *ε*_1_ < 0 and τ→∞. According to Table 4, the values of *ε*_1_ for the first solution are positive, whereas they are negative for the second solution. Consequently, this result demonstrates that the first solutions are physically stable and dependable while the second solutions are not.

## 5. Conclusions

The present study analyzed the effect of Darcy–Forchheimer on nanofluid flow through an exponentially shrinking surface. The governing boundary layer system of the partial equations was reduced into ODEs before solving them by the *bvp4c* technique in MATLAB software. The presence of duality is shown by the numerical results. The stability analysis is conducted to check the stability of the first and second solutions. The numerical value of the smallest eigenvalue in the stability table showed that only the first solution is stable. The coefficient of skin friction increases for the first solution while reducing for the second solution as the values of volume fraction enhance. The rate of heat transfer decreases for the first and second solutions with the increase in the volume fraction values. The variation of the velocity profile decreases/increases in both solutions as the magnitude of the Forchheimer parameter values increases.

## Figures and Tables

**Table 1:** Thermophysical properties of nanofluid [44].

Properties	Nanofluid
Dynamic viscosity	$\mu_{nf}=\frac{\mu_{f}}{{\left(1-\phi \right)}^{2.5}}$
Density	$\rho_{nf}=\left(1-\phi \right)\rho_{f}+\phi \rho_{s}$, where subscript *s* indicates the solid properties of the copper.
Thermal conductivity	$k_{nf}=\frac{k_{s}+2k_{f}-2\phi \left(k_{f}-k_{s}\right)}{k_{s}+2k_{f}+\phi_{s}\left(k_{f}-k_{s}\right)}\times \left(k_{f}\right)$
Heat capacity	${\left(\rho c_{p}\right)}_{nf}=\left(1-\phi \right){\left(\rho c_{p}\right)}_{f}+\phi {\left(\rho c_{p}\right)}_{s}$

**Table 2:** Water and copper thermo-physical characteristics [11].

Material	*ρ*/(kg·m^−1^)	*C_p_*/(J·kg^−1^·m^−1^)	*k*/(W·m^−1^·K^−1^)
Copper	8933	385	401
Water	997.1	4179	0.613

**Table 3:** Magnitude of *f*″(0) and −*θ*(0) under various estimates of *S*.

	Waini et al. [56]		Current Results	
*S*	*f*″(0)	−*θ*(0)	*f*″(0)	−*θ*(0)
0	−1.28181	4.97911	−1.28181	4.97911
0.2	−1.37889	5.65473	−1.37889	5.65473
0.6	−1.59824	7.22487	−1.59824	7.22487
1	−1.84983	9.03715	−1.84983	9.03715

**Table 4:** Smallest eigenvalues for different values of *S* and ∅.

		1st Solution	2nd Solution
*S*	∅	*ε*_1_	
2.5	0.01	0.78456	−0.94592
	0.1	0.64948	−0.89248
3	0.05	0.85310	−0.894601
	0.1	0.68092	−0.87253

## Data Availability

The datasets used and/or analyzed during the current study are available from the corresponding author (A.A.) upon reasonable request.

## Nomenclature

*F_S_*	Forchheimmer parameter
*K*_1_	permeability parameter
*N_u_*	Nusselt number
l*T*_∞_	ambient temperature (*Kelvin*)
*Re_x_*	Reynolds number
*v_w_*	suction/injection velocity (m/s)
*δ_T_*	thermal slip
*μ_nf_*	dynamic viscosity of nanofluid (kg/ms)
*ρ_nf_*	density of nanofluid(kg/m^3^)
′	differentiation with respect to *η*
*M*	Hartmann number
*B(x)*	magnetic field (*Tesla*)
*Pr*	Prandtl number
*T*	temperature (*Kelvin*)
*S*	suction/blowing parameter
*K*	permeability of the porous medium
*u,v*	velocity components (m/s)
*ϕ*	volume fraction of copper
*ε*	unknown eigenvalue
*τ*	stability transformed variable
*T_w_*	variable temperature of sheet (*Kelvin*)
*ε*_1_	smallest eigenvalue
*T*_0_	a constant
*u_w_*	shrinking velocity of surface (m/s)
*η*	transformed variable
*D*	thermal slip factor
*σ*	electrical conductivity (S/m)
*t*	time (s)
*B*	velocity slip factor
*ψ*	stream function
*δ*	velocity slip
*C_f_*	skin-friction coefficient
*B*_0_	constant magnetic strength
*b*	local inertia coefficient
*Ec*	Eckert number
*k_nf_*	nanofluid thermal conductivity (W/mK)
